# Fructans Prime ROS Dynamics and *Botrytis cinerea* Resistance in *Arabidopsis*

**DOI:** 10.3390/antiox9090805

**Published:** 2020-09-01

**Authors:** Henry Christopher Janse van Rensburg, Zoltan Takács, Florentina Freynschlag, Ebru Toksoy Öner, Claudia Jonak, Wim Van den Ende

**Affiliations:** 1 Laboratory of Molecular Plant Biology, KU Leuven, Kasteelpark Arenberg 31, 3001 Leuven, Belgium; henry.jansevanrensburg@kuleuven.be; 2 AIT Austrian Institute of Technology, Center for Health & Bioresources, Bioresources, Konrad Lorenz Strasse 24, 3430 Tulln, Austria; Zoltan.Takacs@ait.ac.at (Z.T.); Florentina.Freynschlag@ait.ac.at (F.F.); Claudia.Jonak@ait.ac.at (C.J.); 3 IBSB, Industrial Biotechnology and Systems Biology Research Group, Bioengineering Department, Marmara University, 34722 Istanbul, Turkey; ebru.toksoy@marmara.edu.tr

**Keywords:** fructan, *Botrytis cinerea*, *Arabidopsis thaliana*, priming, reactive oxygen species, sweet immunity, sugars

## Abstract

Naturally derived molecules can be used as priming or defense stimulatory agents to protect against biotic stress. Fructans have gained strong interest due to their ability to induce resistance in a number of crop species. In this study, we set out to establish the role of fructan-induced immunity against the fungal pathogen *Botrytis cinerea* in *Arabidopsis thaliana*. We show that both inulin- and levan-type fructans from different sources can enhance *Arabidopsis* resistance against *B. cinerea*. We found that inulin from chicory roots and levan oligosaccharides from the exopolysaccharide-producing bacterium *Halomonas smyrnensis* primed the NADPH-oxidase-mediated reactive oxygen species (ROS) burst in response to the elicitors flg22, derived from the bacterial flagellum, and oligogalacturonides (OGs), derived from the host cell wall. Neither induced a direct ROS burst typical of elicitors. We also found a primed response after infection with *B. cinerea* for H_2_O_2_ accumulation and the activities of ascorbate peroxidase and catalase. Sucrose accumulated as a consequence of fructan priming, and glucose and sucrose levels increased in fructan-treated plants after infection with *B. cinerea*. This study shows that levan-type fructans, specifically from bacterial origin, can prime plant defenses and that both inulin and levan oligosaccharide-mediated priming is associated with changes in ROS dynamics and sugar metabolism. Establishing fructan-induced immunity in *Arabidopsis* is an important step to further study the underlying mechanisms since a broad range of biological resources are available for *Arabidopsis*.

## 1. Introduction

The growth and development of plants is adversely affected by external factors that can be divided into abiotic (water limitation, extreme temperatures, salt stress etc.) and biotic stresses (microbial pathogens, insects and viruses). To survive and propagate, it is important for plants to successfully adapt and respond to these stresses [1]. Plants contain several constitutive defenses, which are always present and offer a continuous protection against pathogens [2]. When this line of defenses is breached, plants induce their chemical defenses known as inducible defenses. The majority of responses are induced upon the perception of stimuli, and are thus more specific than constitutive defenses [3]. As they are produced only when required, induced defenses are considered a more efficient allocation of resources that increases the benefit-to-cost ratio to the plant [4]. Upon the recognition of these stimuli, better known as elicitors, several defense responses are activated. Recognition of elicitors is usually also accompanied by a rapid and transient production of reactive oxygen species (ROS) in the apoplast acting as both antimicrobial agents and signaling molecules [5,6]. The necrotrophic fungus *Botrytis cinerea* can infect more than 500 plant species and causes significant damage to both plants and fruits estimated at over USD 100 billion worldwide [7]. Necrotrophs like *B. cinerea* can be negatively affected by an early production of ROS, but also benefit from over-accumulation of ROS during the later stages of infection [8,9].

Several naturally occurring compounds are able to “prime” plant defenses by inducing a physiological status that allows plants to respond more effectively to subsequent abiotic and/or biotic stresses [10,11]. The process of treating plants with such compounds is known as priming. It is proposed that priming involves minimal energy cost and gene induction, and does not induce defense responses directly, but rather promotes a better perception and amplification of stress responses later on [12]. Priming compounds are very diverse, including organic acids, amino acids and host-derived compounds produced as a consequence of (pathogen-inflicted) damage [11,13,14]. One of the best characterized immune elicitors or microbe-associated molecular patterns (MAMP) in plants is the bacterial peptide flg22, derived from the bacterial flagellum [15]. Another elicitor from host origin or damage-associated molecular patterns (DAMPs) in plants is oligogalacturonides (OGs), derived from the breakdown of plant cell-wall fragments [16]. As a first line of defense, both flg22 and OGs are perceived in the apoplast by membrane bound receptors activating a cascade of defense responses [15,16]. Carbohydrates are among the more recently characterized interesting priming agents, mainly due to their low cost of production and ready availability [17,18]. Fructans, polysaccharides mainly consisting of 5-membered fructose (Fru) rings, specifically inulin from plant origin, like those derived from chicory (*Cichorium intybus*) and burdock (*Arctium lappa*), are able to induce resistance against *B. cinerea* in lettuce [19]. Recently, xylo-oligosaccharides with 5-membered arabinose side branches were shown to act as DAMPs and elicit immune responses in plants [20]. Hitherto, β-aminobutyric acid (BABA), chitosan and OGs are among the most popular environmentally friendly agents to induce disease resistance in the agronomical context, next to the widespread use of biological control organisms and their culture filtrates (e.g., *Trichoderma* sp.) [21,22,23,24].

Apart from being central to plant metabolism, simple sugars such as glucose (Glc) and sucrose (Suc) also function as signaling entities during growth and development [25,26] and stress responses [27,28]. The “sweet immunity” concept proposes that extracellular and intracellular sugar dynamics, and intermediates in their metabolism, play an integral part in defense strategies of plants [29]. While structurally derived carbohydrates such as cellobiose and OGs are well-known priming agents, knowledge on non-structural carbohydrates or soluble sugars is scarce [18,30]. It has been shown that exogenous fructans (inulins derived from burdock: burdock fructo-oligosaccharides, BFOs) have the ability to increase resistance of tomato and tobacco plants against *B. cinerea* and tobacco mosaic virus, respectively [31,32]. It is well known that fructan accumulating plants are able to withstand freezing and subsequent cellular damage, suggesting that fructans may act as DAMPs during abiotic stress responses [33]. Fructans with a short degree of polymerization (DP) also accumulate in the apoplast during extended periods of freezing [34]. Besides acting as DAMPs in fructan accumulators, fructans, and specifically levans, may also be perceived as MAMPs in higher plants, giving the fact that several microorganisms accumulate levan-type fructans [35]. This makes fructans particularly interesting compounds to be used as priming agents to induce broad spectrum resistance against abiotic and biotic stresses.

Fructans are formed by the addition of Fru moieties to the fructosyl part of Suc, forming β(2-1) and/or β(2-6) linkages, discriminating the two main fructan types, namely, inulin (β(2-1) and levan (β(2-6)), among other types including graminans, agavins and neokestose-derived fructans [36]. They occur in about 15% of flowering plants, and their accumulation is typically associated with tolerance against abiotic stresses such as drought and cold [37,38]. There are also a few reports on fructan accumulation during biotic interactions [39,40,41], but their function during these interactions requires further research.

A major hindrance in understanding the mechanisms at the base of fructan-induced immunity is the limited availability of genetic toolsets in the crops studied so far. Thus, to set the stage for detailed investigations on the underlying signaling pathways, we investigated whether fructan-induced defense responses are conserved in the non-fructan accumulator model plant *Arabidopsis thaliana* for which a large number of mutants are available. We screened several inulin-and levan-type fructans for their ability to induce resistance in *Arabidopsis* against *B. cinerea*, a major fungal pathogen. The most promising fructans were then used to study their effect on ROS dynamics and soluble sugars during priming and subsequent *B. cinerea* infection. 

## 2. Materials and Methods 

### 2.1. Plant Material and Growth Conditions

*Arabidopsis thaliana* (Col-0) seeds were transferred to square pots (9 × 9 × 8 cm), 5 plants per pot, in a mixture of potting soil and vermiculite (3:2) after a 3-day stratification at 4 °C in the dark. Plants were grown in a Conviron^®^ (Berlin, Germany) growth chamber under 12 h light (21 °C) and 12 h dark (18 °C) light cycle under cool-white fluorescent lamps with 100 μmol m^−2^ s^−1^ light intensity and 60% relative humidity. Plants used in this study were all between 4 to 5 weeks old.

### 2.2. B. cinerea Cultivation and Preparation

*B. cinerea* strain B05.10 was kindly provided by Prof. Barbara De Coninck (KU Leuven, Division of Crop Biotechnics, Leuven, Belgium). For spore production, *B. cinerea* spores from glycerol stocks were germinated and grown on 24 g/L potato dextrose agar (PDA) plates for 14 days at 21 °C in the dark. Spores were harvested with sterile 0.0001% Tween-20 ddH_2_O by scraping the surface with a 1 mL pipette tip. Finally, spores were filtered through a filter consisting of glass wool to remove the mycelia. Spores were allowed to hydrate at 4 °C overnight. Spore concentration was determined using a Neuhauer hemocytometer and light microscope. Spores were adjusted to a concentration of 1 × 10^5^ spores/mL in sterile 12 g L^−1^ potato dextrose for the infection buffer. Infection control (IC) buffer contained 12 g L^−1^ potato dextrose without spores. Spores were inoculated at room temperature for 4 h in the infection buffer before performing infections to allow synchronous germination.

### 2.3. Preparation of Priming Compounds 

Inulin from chicory roots was purchased from Sigma-Aldrich (St. Louis, MO, USA) as a powder. Similarly, p95, a product derived from inulin treatment with an endo-inulinase, was purchased from Beneo-Orafti^®^, Tienen, Belgium. BFOs were extracted from burdock roots as described previously [19]. Levan was kindly provided by Prof. Ebru Toksoy Öner (Industrial Biotechnology and Systems Biology Research Group, Department of Bioengineering, Marmara University, Istanbul, Turkey) and was produced enzymatically using a recombinant levansucrase from *Halomonas smyrnensis* AAD6^T^ as previously described [42]. Levan oligosaccharide (LOS) was produced by enzymatic breakdown of *Halomonas smyrnensis* levan using a purified endo-levanase (LevB) from *Bacillus subtilis* [43]. Briefly, 1 g levan was incubated with purified LevB enzyme in 40 mL 50 mM phosphate buffer (pH 6.1) at 30 °C for several days. Reaction was then boiled at 90 °C for 5 min and centrifuged for 10 min at 7800 RCF (4 °C). Supernatant was transferred to a new tube and longer DP levan was removed by precipitation with 80% acetone and centrifugation for 10 min (4 °C) at 7800 RCF. The supernatant was transferred to a new tube and subsequently evaporated using a rotary evaporator to remove acetone. The resulting pellet was resuspended in ddH_2_O and passed through a Dowex 50WX8 H^+^ and Dowex 1X8 100–200 mesh Ac^−^ (Sigma-Aldrich) column. The pH of the flow-through was adjusted to pH 7.0 using sodium bicarbonate followed by freeze-drying using a lyophilizer (LSL Secfroid, Aclens, Switzerland). 

*Dactylis* levan was produced by floating 200 g of *Dactylis glomerata* leaves in 100 mM Suc for 48 h under continuous light to induce levan accumulation [44]. Pigments were removed by incubating leaves with 800 mL absolute ethanol for 10 min at 80 °C. Leaves were then extracted by incubation in 800 mL ddH_2_O at 80 °C for 15 min. The extract was passed through a cheesecloth, and pH was adjusted to pH 11 using Ca(OH)_2_ as part of the liming process. Subsequently, the pH was adjusted to pH 8.0 using gaseous CO_2_ perfusion in the carbonation process. Calcium carbonate produced was removed by centrifugation at 8000× *g* for 10 min. Liming and carbonation procedures were then repeated twice. Extract was then concentrated using a rotary evaporator and passed through a Dowex column as explained previously. The column was washed with 3 column volumes of ddH_2_O, and the flow through was concentrated using a rotary evaporator. Levans were precipitated using 60% acetone and washed three more times with 60% acetone. Precipitate was freeze-dried using a lyophilizer. A chromatogram representing the High-Performance Anion-Exchange Chromatography with Integrated Pulsed Amperometric Detection (HPAEC-IPAD) (for description see Section 2.9) profiles for all the different fructans can be found in Figure S1.

### 2.4. Plant Treatments 

*Arabidopsis* plants of 4 to 5 weeks old were randomly numbered and separated into the desired treatment groups. Plants were well watered 1 h before performing treatments. All treatments were prepared in ddH_2_O containing 0.0001% Tween-20 (Acros organics) as surfactant. The H_2_O control treatment contained only ddH_2_O supplemented with 0.0001% Tween-20 as to represent conditions of the test substances. Plants were treated by gently spraying them at a distance of ±15 cm away using a spray bottle. Each pot, containing 5 plants, was evenly sprayed with 5 mL solution so that the entire surface of the plants was covered. Untreated plants were handled alongside the treatments, but without being sprayed. Plants were again randomly distributed and placed back in the growth chamber. 

### 2.5. B. cinerea Infection and Disease Scoring

For *B. cinerea* infection, the detached leaf approach was followed as explained previously with minor modifications [45]. Briefly, 72 h after performing treatments, 3 source leaves (rosette leaves 5–7) per plant were harvested using scalpel blade and forceps and rinsed in sterile ddH_2_O to remove residual treatment. Leaves were gently blotted with paper towel to remove water droplets, and placed adaxial side upward in a square petri dish (Greiner Bio-One, Frickenhausen, Germany) lined with a moist paper towel to maintain humidity. Each leaf was inoculated on the tip of the leaf with a single five µL droplet of infection buffer with or without spores (IC). Plates were covered with lids, sealed with parafilm, and placed in an infection room maintained at 18 °C and a 12 h light/12 h dark light cycle. Disease progression was analyzed by taking photos 72 h after infection, and the necrotic lesion area was measured using a 1 cm² reference and the software ImageJ 1.5T (https://imagej.nih.gov/ij/). 

### 2.6. ROS Burst Measurements

The ROS burst was carried out using a luminol-based assay according to the protocol of Albert et al., 2015, with minor modifications. Plants were treated as explained previously and placed back in the growth chamber. Leaf disks were punched at 16 h (6 h after priming) from the source leaves (similar to infected leaves) of plants using a 3.5 mm cork-borer and a plastic rack with holes slightly larger than the cork-borer. Leaf disks were then transferred to a white flat-bottom 96-well plate (Greiner Bio-One) containing 150 µL sterile ddH_2_O, covered with aluminum foil and incubated at 21 °C for 16 h to recover. The water was then replaced with 100 µL incubation solution containing 20 µM luminol L-012 (Wako Chemicals, Richmond, VA, USA), and 1 µg/mL horseradish peroxidase (AppliChem, Darmstadt, Germany) prepared in ddH_2_O. Background luminescence was measured immediately for 30 min using a GloMax^®^-Multi Detection System (Promega, Madison, WI, USA). Using a multipipette, 100 µL of assay buffer consisting of incubation solution and elicitor (at double the final concentration) was then added to start the reaction. For flg22, a final concentration of 100 nM [46] was used and for OGs a concentration of 0.2 mg/mL [47]. OGs were prepared as described previously [19]. For NADPH-oxidase inhibitor assays, diphenyleneiodonium (DPI) was added to both incubation and assay solutions to a final concentration of 5 µM. For the direct effect of fructans on the ROS burst, elicitors were replaced by either inulin or LOS at concentrations of 0.05, 0.5, and 5 mg/mL in the assay solution. Luminescence was then measured for 60 min at 2 min intervals and a 0.5 s integration time. Luminescence readout is given as relative light emitting units (RLU). Background readings were subtracted from the samples to obtain the elicitor-induced response.

### 2.7. H_2_O_2_ Extraction and Quantification

Extraction and quantification of H_2_O_2_ were performed using the eFOX method according to Cheeseman, 2006 with slight modifications [48]. Two source leaves from two different plants were flash-frozen in liquid nitrogen for each replicate, and samples were immediately extracted to prevent loss of H_2_O_2_ during storing. Frozen leaves were grinded in 20 volumes ice-cold 5% trichloroacetic acid (TCA) using a pre-cooled mortar and pestle. Extracts were then centrifuged for 30 min (4 °C) at 15,000× *g* to remove plant debris, and 500 µL of supernatant was added to 500 µL of eFOX reagent (200 µM xylenol orange (Honeywell Fluka, Steinheim, Germany), 500 µM ferrous ammonium sulphate (Honeywell Fluka^TM^), 200 mM sorbitol and 1% EtOH prepared in 50 mM H_2_SO_4_). Reactions were incubated for at least 30 min before measuring the absorbance at 550 nm and 800 nm using a spectrophotometer (Spectronic Genesys 5, Thermo Scientific Inc, Waltham, MA, USA). The concentration of H_2_O_2_ was calculated using a standard curve prepared similar to samples but using dilutions of 30% H_2_O_2_ (Sigma) in the range between 0 to 200 µM instead of plant extract. 

### 2.8. Antioxidant Enzyme Extraction and Activity Measurements

Antioxidant enzymes were extracted according to Yang et al., 2011 with minor modifications [49]. Enzymes were extracted by adding 300 µL extraction buffer (100 mM phosphate buffer, pH 7.0, 0.1% Triton X-100, 15% glycerol, 1 mM Phenyl Methyl Sulphonyl Fluoride, 1 mM ascorbic acid and 0.35 mM β-mercaptoethanol) to 100 mg grinded plant material and homogenized on ice using a micro pestle in a 1.5 mL Eppendorf tube for 30 s. Samples were then vortexed and centrifuged at 10,000× *g* (4 °C) for 15 min. Extracts were stored at −80 °C until used in assays, except for ascorbate peroxidase (APX) extracts which were measured immediately.

Catalase (CAT) activity was measured using a modified protocol by Gil-ad et al., 2000 [50] and Yang et al., 2011 [49], measuring the breakdown of H_2_O_2_ spectrophotometrically. Activity was measured by adding 40 µL of the previously extracted enzyme to 2 mL of assay buffer (100 mM phosphate buffer (pH 7.0)). Background was measured for 30 s before adding 40 µL of 1 M H_2_O_2_ to start the reaction. After 10 s, the catalase-mediated breakdown of H_2_O_2_ was measured at an optical density (OD) of 240 nm for 5 min at 10 s intervals using a Spectronic Genesys 5 Spectrophotometer (Thermo Scientific). A second blank consisted of assay buffer and H_2_O_2_ without enzyme. 

APX was measured by following the breakdown of ascorbic acid as explained previously [51]. Reactions were performed by adding 25 µL enzyme extract to assay buffer containing 1.840 mL 100 mM phosphate buffer (pH 7.0) and 0.5 mM ascorbic acid. The background (blank) was measured for 1 min to correct for non-specific ascorbate breakdown before adding 100 µL 27 mM H_2_O_2_ to start the reaction. Reactions were followed spectrophotometrically at an OD of 290 nm for 5 min at 10 s intervals. A second blank consisted of assay buffer with the addition of H_2_O_2_ but no enzyme. 

Guaiacol peroxidase (GPX) was measured by following the oxidation of guaiacol spectrophotometrically as explained previously [52]. Enzyme (25 µL) was added to the assay buffer (1.875 mL 100 mM phosphate buffer (pH 7.0) and 1 mL of 25 mM guaiacol) and background activity measured for 1 min before adding 100 µL of 2% H_2_O_2_ to start the reaction. The increase in OD_480_ was measured for 5 min at 10 s intervals. The second blank consisted of assay buffer and H_2_O_2_ without the addition of enzyme.

For all enzymes, the activity was calculated from the linear range of the reaction and expressed as units (U)·mg protein^−1^, where 1 U is equal to a 0.01 change in OD value per min. Protein concentration was determined using the Bradford method and a standard curve prepared using Bovine Serum Albumin (Sigma) [53].

### 2.9. Soluble Sugar Extraction and Quantification 

Soluble sugars were extracted in ddH_2_O by boiling as previously described [54]. Briefly, samples (source leaves) were grinded to a fine powder in liquid nitrogen, and 100 mg of plant material was extracted in 1 mL ddH_2_O by boiling at 95 °C for 15 min in a water bath. Samples were allowed to cool down before centrifuging for 10 min at 15,000× *g* to remove plant debris. The supernatant (200 µL) was transferred to a Dowex^®^ anion and cation exchange column, and the column washed 6 times with 200 µL ddH_2_O to remove residual sugars. Flow-through was diluted 1:1 in 20 µM rhamnose H_2_O (used as internal standard) before analysis. Sugars were measured by injecting 6 µL into a HPAEC-IPAD Dionex 5000 (Thermo Scientific, Waltham, MA, USA) with separation on a CarboPac^TM^ PA100 column (Thermo Scientific, Waltham, MA, USA) and a mobile phase of 90 mM NaOH. Concentrations were calculated using standards of 10 µM of each sugar ran alongside.

### 2.10. Graphical Preperation and Statistical Analysis 

Graphs were prepared using GraphPad Prism version 8.0.0 for Windows, GraphPad Software, San Diego, CA USA, www.graphpad.com and Inkscape 1.0 (Free Software Foundation). Statistical analysis was performed using GraphPad Prism version 8.0.0 for Windows, GraphPad Software, San Diego, CA USA, www.graphpad.com.

## 3. Results

### 3.1. Inulin- and Levan-Type Fructans Induce Resistance against B. cinerea

**Figure 1:**
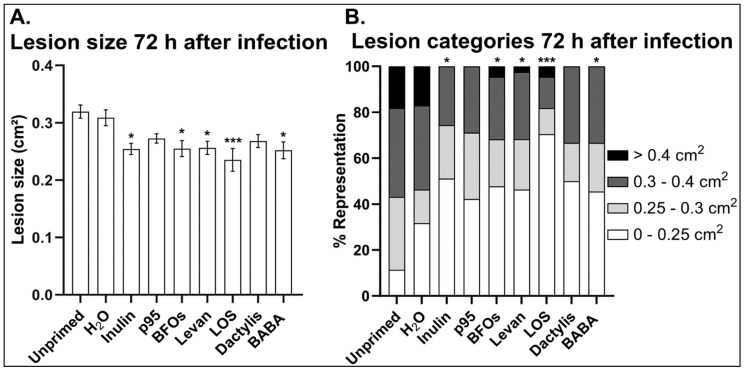
(**A**) Average lesion size of fructan-treated (5 g/L) plants compared to untreated and H_2_O controls. β-Aminobutyric acid (BABA) was used as positive control at 1 mM. Bars represent the mean ± SE of at least 40 biological replicates. (**B**) Disease severity of fructan-treated plants grouped into four different classes based on the percentage representation of their lesion sizes. Each treatment contained at least 40 biological replicates and was repeated three times with consistent results. Asterisks indicate statistical significance of the mean and SE against H_2_O treatment using one-way ANOVA followed by Dunnett’s multiple comparison test (* *p* < 0.05, *** *p* < 0.001).

As a first step, we set out to determine whether fructans are able to enhance *Arabidopsis* resistance against *B. cinerea*. For this we screened a range of inulin- and levan-type fructans from different origins for their ability to induce resistance in comparison with BABA, a well-known priming agent (Figure 1). We analyzed two commercially available inulin-type fructans from chicory roots namely inulin (Sigma) and p95 (Orafti^TM^) as well as BFOs extracted from burdock roots. The levans tested were high DP levan from the Gram negative, exopolysaccharide-producing bacterium *Halomonas smyrnensis* (levan), a hydrolyzed fraction thereof with a shorter DP (levan oligosaccharide: LOS), and levan extracted from Suc-induced *Dactylis glomerata* (*Dactylis*) leaves. Chromatograms indicating the profile of each fructan can be found in the Supplementary Materials (Figure S1). A concentration (5 g/L) similar to those previously reported to be effective for BFOs and inulin was used [19]. *Arabidopsis* plants (4–5 weeks old) were leaf-sprayed with the different classes of fructans, and 3 days later the source leaves were detached, rinsed with ddH_2_O and infected with *B. cinerea*. 72 h after infection; lesion sizes were measured and compared to unprimed and H_2_O-sprayed controls. 

In general, all tested fructan types reduced the lesion sizes compared to untreated and H_2_O-treated controls (Figure 1). Seventy-two hours after infection, *Arabidopsis* leaves treated with inulin and LOS showed the smallest lesion development, even smaller than those treated with the positive control BABA (Figure 1A). We used the most effective concentration of BABA (1 mM) determined previously in our leaf priming system [55,56,57]. Spraying with BFOs and high DP levan also led to a reduced lesion development, but to a lesser extent. On the contrary, both p95 and *Dactylis* levan did not induce a significant decrease in lesion size compared to the H_2_O control (Figure 1A). It is interesting to note that when the lesions were separated according to their size, the majority of the *Botrytis*-induced lesions on fructan-treated leaves categorized into the lower two size classes representing lesions smaller than 0.3 cm^2^ with little to no lesions larger than 0.4 cm^2^ (Figure 1B). Remarkably, more than 70% of the lesions of LOS-treated plants were below 0.25 cm^2^, while only about 35% of the lesions of H_2_O-treated leaves fell into this category (Figure 1B). Overall, these data show that fructans can provide comparable protective effects to well established priming agents such as BABA and that LOS derived from *Halomonas smyrnensis* levan is a highly effective priming agent in *Arabidopsis,* outcompeting BABA.

### 3.2. B. cinerea Germination and Growth Is Not Significantly Affected by Fructans

Next, we determined whether the fructans used for treatments have a direct effect on the growth of *B. cinerea*. To test this, we looked at both the germination of spores and hyphal growth on PDA plates supplemented with fructans at 5 g/L (the same concentration used in treatments). To determine the germination efficiency of *B. cinerea* spores, 1 × 10^5^ spores/mL were plated on PDA plates supplemented with fructan and allowed to germinate. After 24 h, the percentage of germinated spores were counted and compared to that on control plates containing no fructan. There was no clear inhibitory effect on spore germination posed by any of the tested fructans (Table 1). *Dactylis* levan slightly but not significantly reduced germination as compared to control. On the contrary, spore germination efficiency was marginally improved on plates supplemented with p95, LOS and high DP *Halomonas* levan (Table 1). 

**Figure 2:**
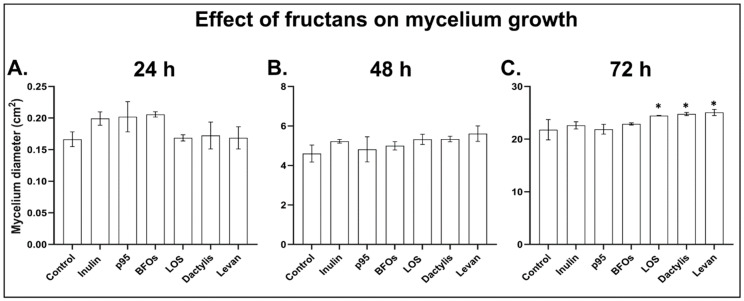
The effect of different fructans on the mycelium growth of *B. cinerea*. Diameter of *B. cinerea* mycelium grown on PDA plates supplemented with 5 g/L fructan after (**A**) 24 h, (**B**) 48 h and (**C**) 72 h. Bars represent the mean ± SE (*n* = 5). Asterisks indicate statistical significance (* *p* < 0.05) according to one-way ANOVA followed by Tukey’s multiple comparisons test and adjusted *p* values.

To establish whether any of the tested fructan types can inhibit the growth of *B. cinerea* directly, we followed the mycelium growth on PDA plates supplemented with fructans. After spores were transferred to plates, the diameter of the mycelial growth was recorded for 3 consecutive days and compared to that on control plates without fructans. None of the tested fructans showed any direct inhibitory effect on *B. cinerea* mycelium growth (Figure 2). On the contrary, most fructans stimulated the growth of *B. cinerea* to some extent. After 72 h the mycelium diameter was significantly larger on plates supplemented with any of the three levan-type fructans as compared to control. There were no clear differences in the mycelium growth phenotype between the different plates (Figure S2). The growth assays correlated well with the germination assays, indicating that fructans neither affect *B. cinerea* germination nor growth. These data together with the observation that fructans enhanced *Arabidopsis* resistance against *B. cinerea* thus suggest that fructans can enhance plant immunity rather than inhibit fungal growth.

### 3.3. Inulin and LOS Prime the flg22 and OGs Elicitor-Mediated ROS Burst

Elicitors such as flg22 and OGs trigger a cascade of signaling events towards the activation of defense responses. The oxidative burst, the accumulation of apoplastic ROS within minutes after elicitor perception, is a hallmark of early defense signaling [58]. On the contrary, priming agents such as BABA do not elicit early defense-related signaling events, but prime the plant for a more robust NADPH-oxidase-dependent production of ROS upon perception of elicitors or pathogens [5]. To determine whether fructans function as a priming agent in plants, we sprayed *Arabidopsis* plants with the different inulin- and levan-type fructans prior to assessing flg22- and OGs -induced ROS burst. 

**Figure 3:**
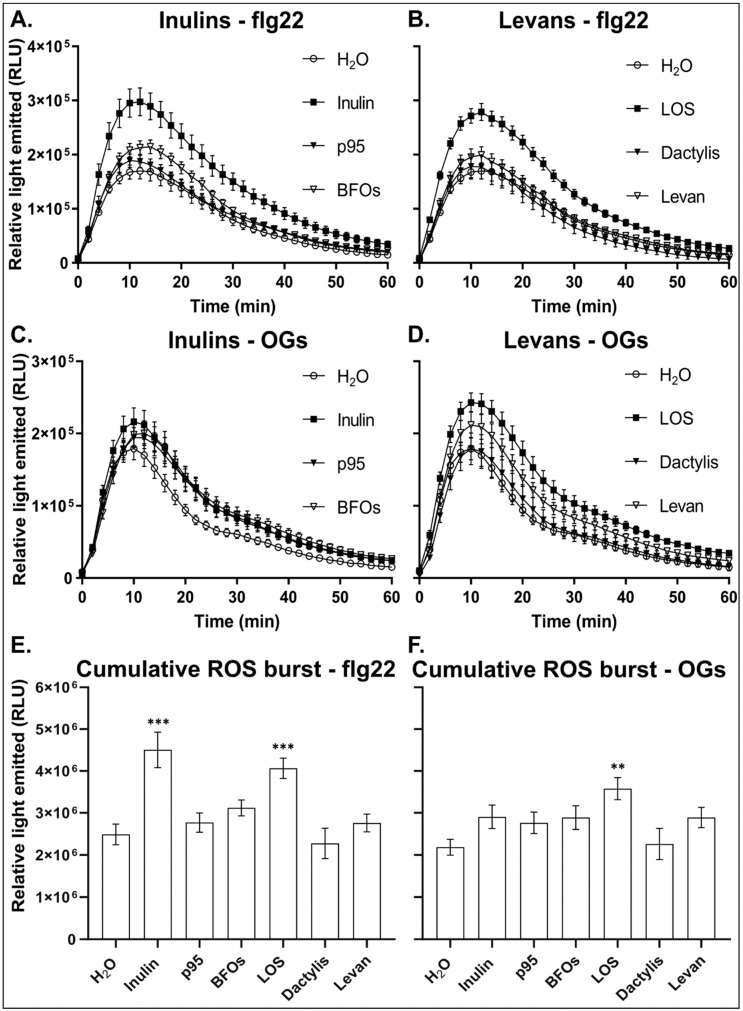
Priming of elicitor-induced reactive oxygen species (ROS) production in plants treated with different fructan types. ROS production in leaf disks in response to 100 nM flg22 or 0.2 mg/mL oligogalacturonides (OGs) 24 h after pre-treatment with 5 g/L inulin-type fructans (**A**,**C**) or levan-type fructans (**B**,**D**). (**E**,**F**) Cumulative ROS produced over 60 min after elicitor treatments. Values are the mean ± SE (*n* = 16) and expressed as relative light units (RLU). Significance is indicated by asterisks (** *p* < 0.01; *** *p* < 0.001) based on one-way ANOVA followed by Dunnett’s multiple comparisons test and adjusted *p* values. Experiment was repeated three times with consistent results.

In response to flg22, leaf disks from inulin and LOS-treated plants generated a significantly higher ROS burst as compared to H_2_O-treated control plants (Figure 3A,B). H_2_O pre-treatment had no significant effect on subsequent flg22- or OG-mediated ROS burst when compared to untreated plants (Figure S3). Levan and BFOs marginally increased ROS production in response to flg22. p95 and *Dactylis* levan had little to no effect. This was also clear when taking into account the cumulative ROS produced (sum of all the timepoints) over the 60 min period after treatment with elicitor (Figure 3E). The cumulative flg22-induced ROS produced during the ROS burst period nearly doubled in plants pre-treated with inulin and LOS as compared to the control (Figure 3E). In comparison, p95, BFOs and levan only showed a marginal increase in the cumulative ROS produced. *Dactylis* levan had no effect on cumulative ROS production. 

In response to OGs, pre-treatment with all inulin-type fructans increased ROS-burst peaks compared to H_2_O control pre-treatments (Figure 3C). Like for flg22, of all inulin-type fructans tested, inulin showed the largest effect on the OG-induced early oxidative burst. The cumulative ROS production was slightly higher than the control (Figure 3F). For levan-type fructans, plants treated with LOS showed a significant increase in both the ROS burst peak and the cumulative ROS production (Figure 3D,F). *Halomonas* levan marginally increased the ROS burst, whereas *Dactylis* levan had no effect (Figure 3D,F). It is interesting to note that inulin and LOS not only showed the highest effect on the flg22- and OG-induced early ROS burst but also had the strongest protective effect against *B. cinerea* infection. 

**Figure 4:**
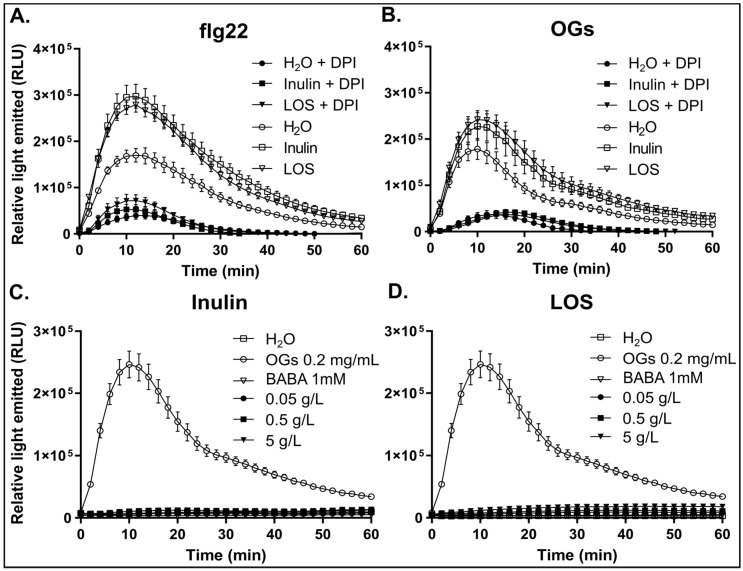
Priming of elicitor-induced NADPH-oxidase-mediated ROS production in plants pre-treated with inulin and LOS (**A**,**B**). ROS production in leaf disks in response to (**A**) 100 nM flg22 or (**B)** 0.2 mg/mL OGs 24 h after pre-treatment with 5 g/L fructan. Diphenyleneiodonium (DPI) was added to 5 µM final concentration for NADPH-oxidase inhibition. The graph represents the mean ± SE (*n* ≥ 8). (**C**,**D**) Absence of a ROS burst in leaf disks in response to inulin and LOS treatment, as compared to BABA, OGs, and H_2_O control treatments with no elicitor. *Arabidopsis* leaf disks were treated with different concentrations of (**C**) inulin and (**D**) LOS and compared to OGs at 0.2 mg/mL and BABA at 1mM. Values represent the mean ± SE (*n* = 16) and expressed as relative light units (RLU).

To test whether the increased ROS burst in response to flg22 and OGs in fructan-primed plants is mediated by NADPH-oxidases, we used the NADPH-oxidase-specific inhibitor, DPI. DPI suppressed the flg22 and OG-induced ROS burst in both primed and non-primed plants (Figure 4A,B), indicating that inulin and LOS pre-treatment enhances the NADPH-oxidase-dependent early oxidative burst in response to elicitor perception. The increased ROS peak is also not related to background ROS production, as the background for each replicate was measured before adding elicitor and subtracted to specifically represent only the elicitor-induced ROS. 

With fructans, specifically inulin and LOS, enhancing resistance against *B. cinerea*, and affecting elicitor-induced responses after plant treatment, we tested whether they also induce a direct response, as commonly observed for typical elicitors such as OGs. Therefore, we performed direct assays on leaf disks using either inulin or LOS in comparison with OGs and BABA to establish their ability to directly induce a ROS burst (Figure 4C,D). While OGs induced a rapid and transient ROS accumulation, neither inulin (Figure 4C) nor LOS (Figure 4D) induced a direct ROS burst. This suggests that fructans, similar to BABA, do not act as a typical elicitor that generate a rapid oxidative burst after perception by the plant. Taken together, the ability of inulin and LOS to enhance the flg22- and OG-induced ROS accumulation without directly inducing a ROS burst further supports the notion that inulin and LOS function as priming agents in *Arabidopsis*.

Based on the strong effect of inulin and LOS on the elicitor-mediated ROS burst, we subsequently focused on these compounds for further analysis on ROS dynamics and sugar levels after priming and during *B. cinerea* infection.

### 3.4. Inulin and LOS Induce ROS Accumulation and ROS-Scavenging Enzymes 

**Figure 5:**
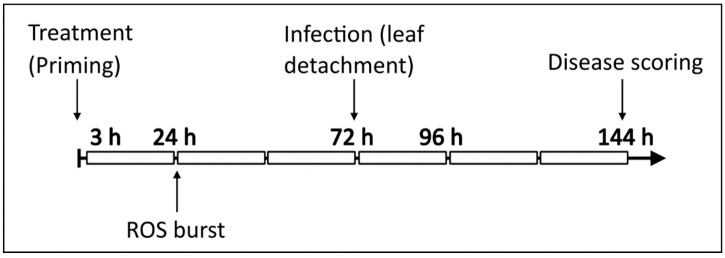
Schematic representation of the experimental setup of priming, infection and sampling of material. Plants were treated with fructans at 0 h. Seventy-two hours after treatments, leaves detached and infected with *B. cinerea*. Samples were taken 3 h, 24 h and 72 h after treatment, and 24 h after infection (96 h after treatment). Disease symptoms were scored 72 h after infection (144 h after treatment). ROS burst experiments were conducted 24 h after treatments.

To better understand the effect of fructan treatment on plants, we explored the H_2_O_2_ levels and the activity of ROS-scavenging enzymes (catalase (CAT), ascorbate peroxidase (APX) and guaiacol peroxidase (GPX)) in the leaves of *Arabidopsis*. For this, plants were treated as previously explained for disease assays and samples were taken at 3 h, 24 h, 72 h (time of infection on detached leaves) and 96 h after treatment (24 h after infection). To discriminate between any effect by the detachment of leaves for infections, we also included controls without spores (IC). Figure 5 illustrates the experimental setup. 

**Figure 6:**
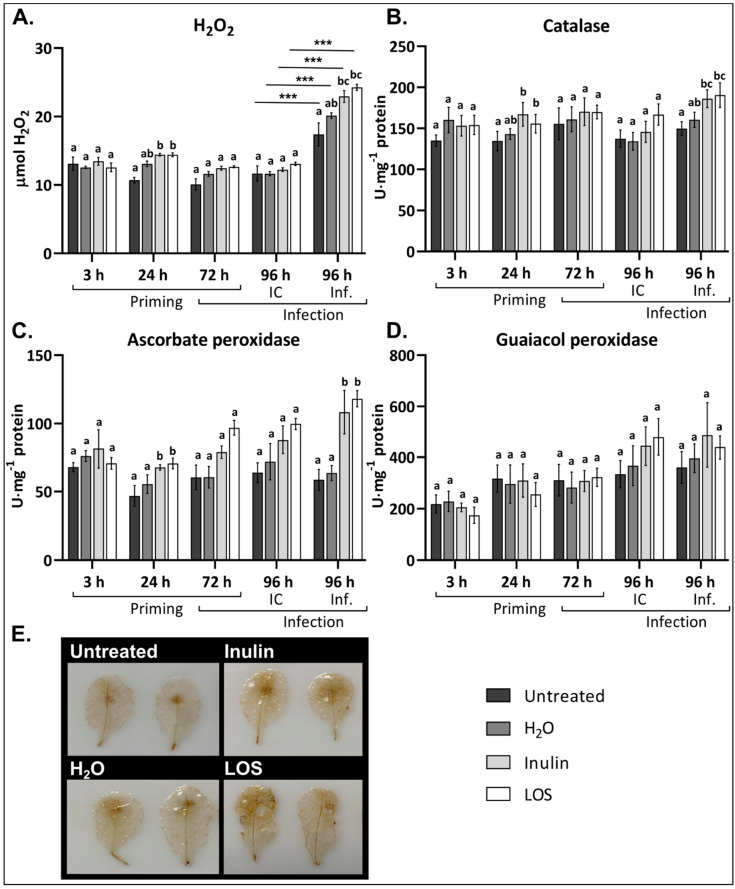
Effect of fructan treatment on H_2_O_2_ content and H_2_O_2_ scavenging enzymes in the leaves after treatment and *B. cinerea* infection. *Arabidopsis* plants treated with 5 g/L inulin or LOS followed by *B. cinerea* infection 72 h later were analyzed for (**A**) H_2_O_2_ content, (**B**) catalase activity, (**C**) ascorbate peroxidase activity and (**D**) guaiacol peroxidase activity. Bars represent the mean ± SE of 6 biological replicates. Statistical significance is indicated by different letters (a–c) within the same timepoint (*p* < 0.05) with asterisks (*** *p* < 0.001) between time points and is based on two-way ANOVA, followed by Tukey’s multiple comparison test and adjusted *p* values. Experiments were repeated three times with consistent results. (**E**) Representative leaves analyzed for H_2_O_2_ distribution by DAB staining 24 h after infection.

Inulin and LOS treatment induced a small but significant transient increase in H_2_O_2_ levels. No difference in H_2_O_2_ content was observed 3 h after fructan treatment. However, 24 h after treatment, both inulin- and LOS-treated plants showed significantly higher ROS levels compared to the untreated control (Figure 6A). Control treatment with H_2_O (containing 0.0001% Tween-20) also induced a marginal but not significant increase in H_2_O_2_ content. The level of H_2_O_2_ was also slightly higher in fructan-treated plants 72 h after treatment, when leaves were detached and infected with *B. cinerea* (as compared to a control without spores). While the IC did not show any changes in H_2_O_2_ content, ROS levels were significantly higher in leaves infected with *B. cinerea* (Figure 6A). Both inulin and LOS pre-treatment led to a significant further increase in H_2_O_2_ levels in *Botrytis*-infected leaves as compared to the untreated and H_2_O controls. In line, DAB (3,3′-diaminobenzidine) staining showed a more pronounced ROS production and distribution in inulin- and LOS-treated leaves (Figure 6E).

Regarding antioxidant enzymes, CAT and APX activity was slightly but significantly increased 24 h after inulin and LOS treatment (Figure 6B,C), following the initial increase in H_2_O_2_ levels at the same time. At the subsequent timepoints, no significant difference in CAT activity was detected in untreated and fructan-treated plants. However, CAT activity was significantly higher in inulin- and LOS-treated leaves 24 h after infection with *B. cinerea* (Figure 6B). Substantial increases in APX activity were detected at all time points after fructan treatment. Interestingly, *Botrytis* infection did not induce APX activity, however, APX activity was significantly increased in inulin- and LOS-treated plants (Figure 6C). While fructans stimulated APX and CAT activity, no significant fructan-induced changes were observed for GPX activity upon pathogen infection. There was also no clear difference in GPX activity between *Botrytis*-infected and uninfected samples (Figure 6D). 

Taken together, both inulin and LOS induced a transient increase in ROS levels and enhanced CAT and APX activity. Upon *Botrytis* infection, H_2_O_2_ levels significantly increased in both untreated and fructan-treated plants. Interestingly, while CAT and APX activity in control plants remained similar in IC and pathogen-infected leaves, both inulin and LOS pre-treatment significantly enhanced CAT and APX activity in leaves infected with *B. cinerea*.

### 3.5. Fructan Treatments Prevent Fluctuating Sugar Levels During Infection

Primary carbon metabolism plays an important role in the integration of diverse environmental and stress signals, and specifically soluble sugars such as Glc and Suc control plant physiology and metabolism as well as stress tolerance [59,60,61]. To determine the effect of fructan treatment followed by *B. cinerea* infection on the soluble sugar content, we used the same batch of leaf samples from inulin- and LOS-treated, untreated and H_2_O-control plants for the H_2_O_2_ and antioxidant enzyme analyses.

**Figure 7:**
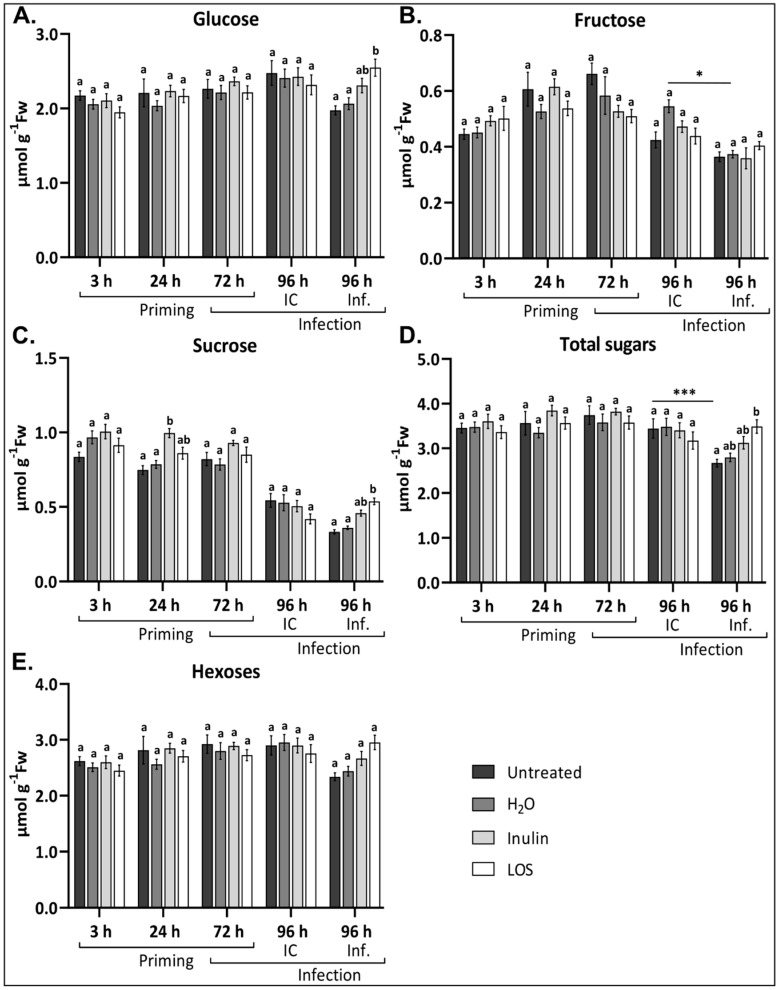
Soluble sugar analysis of the plants (comparable source leaves used in infections) treated with inulin and LOS in response to treatment and *B. cinerea* infection. Quantification of (**A**) Glucose (Glc) (**B**) Fructose (Fru), (**C**) Sucrose (Suc), (**D**) total sugars (Glc + Fru + Suc) and (**E**) hexoses (Glc + Fru) in untreated, H_2_O-treated, inulin-treated and LOS-treated plants (3 h, 24 h and 72 h) followed by infection for 24 h (96 h). Infection control (IC) plants contained infection buffer without spores. Bars represent the mean ± SE (*n* = 6, biological repeats). Statistical significance is indicated by different letters (a,b) within the same time point (*p* < 0.05) and with an asterisk (* *p* < 0.05, *** *p* < 0.001) between time points, and is based on two-way ANOVA followed by Tukey’s multiple comparison test and adjusted *p* values. Experiments were repeated three times with consistent results.

Fructan treatments did not have any significant effect on hexose (Figure 7A,B,E) and total sugar (Figure 7D) levels within the first 72 h. However, Suc levels were significantly higher 24 h and slightly elevated 72 h after inulin and LOS treatments as compared to controls (Figure 7C). After infection with *B. cinerea*, however, significantly elevated levels of Glc, Suc and total sugars were observed for LOS-treated plants and to a lesser extent for inulin-primed plants as compared to controls (Figure 7A,C,D). It is important to note that the significant decrease in Suc content at 96 h in IC and *Botrytis*-infected leaves is attributed to the detachment of the leaves at 72 h, as this is visible in both infected and IC samples irrespective of any treatment (Figure S4A). Interestingly, both Glc and Suc levels were less affected as a consequence of infection in fructan-treated plants as compared to the controls (Figure 7A,C and Figure S4B). Taken together, LOS treatment had a more significant effect on sugar levels than inulin, and a similar trend was observed for ROS dynamics and disease susceptibility.

## 4. Discussion

There is a growing interest in natural and less hazardous approaches to control plant pathogen infections. One of the most intriguing approaches is to harness the full potential of the plants’ innate immune system. Priming the plants immune response using naturally occurring compounds with little to no detrimental effects on the environment is thus gaining increasing interest. As such, readily available carbohydrates with low production cost are highly attractive as potential priming compounds [18]. 

Our data indicate that both inulin- and levan-type fructans can enhance *Arabidopsis* immunity against the necrotroph *B. cinerea*, irrespective of their origin (Figure 1) and without directly inhibiting the germination or growth of *B. cinerea*. However, for unclear reasons, the effects of *Dactylis* levans in the context of the *B. cinerea*/*Arabidopsis* pathosystem were marginal as compared to the recently reported *Venturia inaequalis*/apple pathosystem [62]. Chicory inulin with a higher DP (fructans with terminal Glc) showed higher protective effects as compared to p95, a fructan with a lower DP (mainly fructans without terminal Glc). We demonstrated for the first time that priming with a non-plant-derived levan from *Halomonas* enhanced immunity, and that this protective effect was further increased by enzymatic hydrolysis of this levan (LOS: DP 3–5, mainly fructans without terminal Glc). The lowering of the DP may facilitate entrance into the apoplastic space and increase the interaction with the plasma membrane and embedded immune receptors [63], supporting the hypothesis that fructans act as DAMPs or MAMPs in plants [35]. Fructans of shorter DP also accumulate in the apoplast during periods of freezing, supporting a role for fructans as DAMPs [34]. Fructan perception by animal immune receptors (TLR2 and 4) is well documented [64,65,66]. In *Arabidopsis*, a non-fructan accumulator, fructans are considered to be perceived as MAMPs [35,67]. Endogenous *Arabidopsis* fructan exohydrolases (FEHs) residing in the cell wall/apoplast may be involved in lowering the high DP of microbial fructans, which are usually levans [67]. 

The oxidative burst is associated with the perception of pathogens or elicitors at the plasma membrane and occurs within minutes after their recognition [68,69]. In grapevine, BABA priming leads to an induced expression and activity of NADPH-oxidases in response to OGs treatment [5]. NADPH-oxidases generate the majority of apoplastic ROS produced upon elicitor perception. ROS are proposed to act both as direct antimicrobial and as signaling compounds, specifically apoplastic ROS [5,70,71]. Our data show that fructan pre-treatment induced a higher flg22- and OG-induced ROS burst compared to controls, with inulin and LOS generating the strongest responses (Figure 3), supporting the hypothesis that fructans act as true priming agents. In line with this notion, inulin and LOS did not evoke a direct ROS burst as observed for elicitors (Figure 4C,D). Inhibitor studies suggest that the majority of the flg22- and OG-induced ROS burst may be associated with the apoplast localized NADPH-oxidase activity (Figure 4A,B). Likely, fructan-primed plants may become more sensitive to pathogen-derived endogenous OGs, resulting in a stronger NADPH-oxidase-mediated ROS burst to better counteract necrotrophic pathogens at the early infection stages [68,72]. It has been shown that an active NADPH-oxidase is required for plant resistance against several necrotrophic pathogens [8,9] through antagonized SA signaling limiting programmed cell death (PCD), the latter being able to boost necrotroph spreading [9]. On the other hand, it has been reported that the NADPH-oxidase-mediated oxidative burst is not the critical factor for *Botrytis* resistance during priming with OGs in *Arabidopsis* [47]. In any case, the level of enhanced extracellular ROS observed in plants primed with different fructans correlated with the level of improved resistance against *B. cinerea*. The elevated oxidative burst in inulin- and LOS-treated plants may contribute to signaling to surrounding cells, activate ROS-scavenging systems and prime callose deposition at the site of infection, which is an interesting topic for future studies.

Plant treatment with inulin- and LOS-induced H_2_O_2_ accumulation in the leaves of *Arabidopsis* plants (Figure 6A) supports previous findings regarding a possible role of ROS during fructan priming [19,31]. We also found that inulin- and LOS-treated plants had a significantly higher H_2_O_2_ production in response to *B. cinerea* infection compared to control plants. DAB staining indicated that H_2_O_2_ accumulates in areas further away from the infection site in fructan-treated leaves (Figure 6E). This points towards enhanced long distance ROS/Ca^2+^ waves with ROS diffusing in the apoplast (intercellular signaling) followed by intracellular signaling events and increased NADPH oxidase production during early infection stages in primed plants. 

The activities of enzymes involved in H_2_O_2_ detoxification (CAT and APX) correlated with the level of H_2_O_2,_ both after fructan treatment and in response to infection, preventing ROS from reaching excessive and damaging levels. Interestingly, in inulin- and LOS-treated plants CAT and APX activity significantly and rapidly increased within 24 h after *Botrytis* infection, whereas they remained low in non-primed plants (Figure 6B,C). It is worth noting that APX is considered to be the main ROS-scavenging enzyme during *B. cinerea* infection in tomato plants [73,74]. It has been reported that during *B. cinerea* infection APX activity only increased around 48 to 72 h after infection, suggesting that fructan priming initiates a faster activation of ROS-scavenging enzymes, thus enabling the initiation of a timely plant defense. The unchanged GPX activity after infection might be explained by the fact that cytosolic GPX activity was analyzed (pH 7.0) excluding the apoplastic GPX (slightly acidic pH optimum), and that cytosolic GPX activity has been reported to increase only later after infection [75]. Perhaps the ascorbate-glutathione cycle can also compensate for the low cytosolic GPX at the early infection stages. Further studies are required on the spatio-temporal fluctuations of the ascorbate-glutathione cycle in close association with ROS dynamics in fructan-primed and infected *Arabidopsis* leaves, focusing on the differences between the area of *B. cinerea* infection and the area adjacent to infection [76,77]. 

Taken together with the ROS dynamics observed after fructan priming in case of the *B. cinerea*/lettuce pathosystem [19], all these observations suggest that fructans prime plants for a more robust H_2_O_2_ accumulation and detoxification after infection with *B. cinerea*. These data fit with OG-induced ROS burst assays on plants pre-treated with inulin and LOS, suggesting a possible role for OGs upon *B. cinerea* recognition in fructan-treated plants. Given the complexity of plant defense responses, the cellular ROS status may be one of the factors contributing to fructan-induced immunity.

Soluble sugars and the enzymes that produce them are closely linked to oxidative stress and ROS signaling; however, their effects on the expression of genes result from sugar-specific signaling cascades [78,79]. They also accumulate during several stresses associated with ROS [78]. Although soluble sugars were not strongly affected by fructan priming, an intriguing temporal increase in Suc level was observed (Figure 7C). Temporal Suc accumulation, or changes in the Suc/hexose ratios, are linked to Suc signaling pathways [80,81]. These pathways in turn activate immune responses, stimulating the production of secondary metabolites with antifungal activity [80,81]. Interestingly, fructan treatment, specifically LOS priming, counteracted infection-induced decreases in sugar levels (Figure 6A,C), which might contribute to boost defense responses through Suc signaling events after infection. Intriguingly, it has been shown that BFO treatment restricted the transport of Suc between the mesocarp and exocarp tissue in grapes [82]. In the exocarp, Suc import was inhibited by BFO treatment, leading to delayed senescence. Several Glc and Suc transporters are also induced upon *B. cinerea* infection for the benefit of the pathogen [83,84]. As such, the ability to restrict Suc/Glc transporters might prevent the pathogen from inducing a “sink status” to redirect sugars for its own benefit. Considering our data in this respect, it is possible that fructan priming, particularly LOS priming, might prevent *B. cinerea* to hijack sugar transporters/metabolism, limiting access to soluble sugars. Alternatively, higher sugar levels in fructan-treated plants might be associated with a lower colonization by *B. cinerea,* and thus a reduced sugar consumption by the pathogen.

Previous data revealed that the level of hexoses was retained after inulin-primed lettuces were infected with *B. cinerea* [19]. We observed a similar trend for total sugars and for hexoses, especially in LOS-treated *Arabidopsis* plants. This suggests that these plants can maintain functional metabolic activity to support a pro-survival strategy under necrotrophic attack which favors PCD. Higher sugar levels might also fuel cellular processes required to produce compounds to defend against pathogen invasion. In the case of γ-aminobutyric acid (GABA), a priming against *B. cinerea* for instance, plants are stimulated to maintain normal metabolic activity to provide substrates for pathways, such as the TCA cycle, which are a crucial part during stress responses [85]. Furthermore, sugars such as Glc also serve as the direct building blocks for antioxidants such as ascorbic acid important for the detoxification of excessive pathogen-derived ROS generated during necrotrophic stages of *Botrytis* infection [86].

## 5. Conclusions and Perspectives

Taken together this study shows that a broad range of fructans can enhance *Arabidopsis* resistance to *B. cinerea*. We also showed for the first time that levan-type fructans from bacterial origin can induce plant innate immunity. Both plant-derived inulin and bacterial-derived LOS not only primed the flg22- and OG-mediated ROS burst but also H_2_O_2_ accumulation after *B. cinerea* infection. Fructan priming stimulated the activities of CAT and APX directly after treatment and upon infection with *B. cinerea*. Soluble sugar levels were also affected by fructan priming, specifically after infection. This study demonstrates that fructan priming is effective even in a non-fructan accumulator such as *Arabidopsis*, offering avenues for deeper mechanistic studies. For instance, testing the effect of fructans on plants lacking a functional respiratory burst oxidase homolog D (RBOHD) (NADPH-oxidase), or the use of NADPH-oxidase inhibitors during priming and/or infection will provide further evidence for the role of ROS during primed resistance. Since RBOHD is also a critical player during endoplasmic reticulum stress recovery [87], linked to the unfolded protein response (UPR) and autophagy [88,89], it would be interesting to test fructan priming efficacy in other mutants that are specifically affected in these signaling pathways. For instance, testing mutants defective in UPR activation (inositol-requiring protein-1) or autophagy (AUTOPHAGY5) initiation can unravel whether fructan-treated plants are able to cope more effectively with endoplasmic reticulum stress. Although we found a clear correlation between fructan priming and ROS dynamics, it is important for future studies to determine whether ROS is directly involved in fructan-induced immunity or rather a consequence. It would be interesting to study the transport of specific sugars between subcellular compartments and tissues during priming and infection to improve our knowledge on why sugar levels remain unaffected in primed plants after infection. Furthermore, it would be interesting to compare the effects of leaf spraying with those of vacuum infiltration in a lab setting. The latter approach may provide better tissue accessibility, potentially increasing the amount of fructans that are able to interact with potential membrane receptors that are involved in immune responses. Retrieving deeper molecular mechanistic insights in *Arabidopsis* will be helpful to develop suitable fructan-based priming formulations for crop plants. In conclusion, this study sets the stage for future research to better understand the mechanisms at the base of fructan priming, specifically in *Arabidopsis* where a large number of mutant lines are available. We anticipate an intense cross-talk between sugar, hormone and ROS signaling in these processes. In lettuce, for example, ethylene signaling and ROS dynamics were shown to be involved in inulin-dependent resistance against *B. cinerea* [19]. Detailed studies focusing on the involvement and integration of various endogenous factors offer intriguing perspectives for future studies on the overall role of fructans during stress tolerance. 

## Figures and Tables

**Table 1:** Germination efficiency of *B. cinerea* spores in response to different fructans. Germination efficiency (%) of *B. cinerea* spores 24 h after transfer to 24 g/L potato dextrose agar (PDA) plates supplemented with 5 g/L fructan, or control plates containing PDA only. At least 120 spores from three independent plates were considered for each treatment.

	Control	Inulin	p95	BFOs	Levan	LOS	Dactylis
**% Germination**	89.11	89.32	92.29	89.59	93.51	92.89	81.70
**SD**	2.82	1.65	0.99	1.44	4.97	1.28	3.79

## Supplementary Materials

Supplementary Materials can be found at https://www.mdpi.com/2076-3921/9/9/805/s1. **Figure S1.** Chromatograms resulting from fructan analysis on HPAEC-IPAD. **Figure S2.** *B. cinerea* growth on PDA plates supplemented with fructans. *B. cinerea* grown for 72 h on 24 g/L PDA plates containing 5 g/L fructan. **Figure S3.** (**A**) flg22- and (**B**) OG-induced ROS burst in untreated and H_2_O-treated plants. **Figure S4.** (**A**) Decrease in Suc content as a consequence of leaf defoliation after 24 h. (**B**) Effect of fructan priming on maintaining Suc homeostasis after infection.

## Abbreviations

APX	Ascorbate peroxidase
BABA	β-Aminobutyric acid
BFO	Burdock fructooligosaccharide
CAT	Catalase
DAB	3,3′-Diaminobenzidine
DAMP	Damage Associated Molecular Pattern
DPI	Diphenyleneiodonium
Fru	Fructose
GABA	Gamma aminobutyric acid
Glc	Glucose
GPX	Guaiacol peroxidase
IC	Infection control
LOS	Levan oligosaccharide
MAMP	Microbe Associated Molecular Pattern
OG	Oligogalacturonides
PDA	Potato dextrose agar
RLU	Relative Light Units
ROS	Reactive oxygen species
SA	Salicylic acid
Suc	Sucrose
