# Supplementary material for: Fructans Prime ROS Dynamics and Botrytis cinerea Resistance in Arabidopsis
Source: Antioxidants (Basel). 2020 Sep 1;9(9):805. doi: 10.3390/antiox9090805 (PMC7555011; doi:10.3390/antiox9090805)
Supplement: Supplementary file 1 [file antioxidants-09-00805-s001.pdf]

Supplementary Materials: Supplementary materials can be found at www.mdpi.com/xxx/s1.

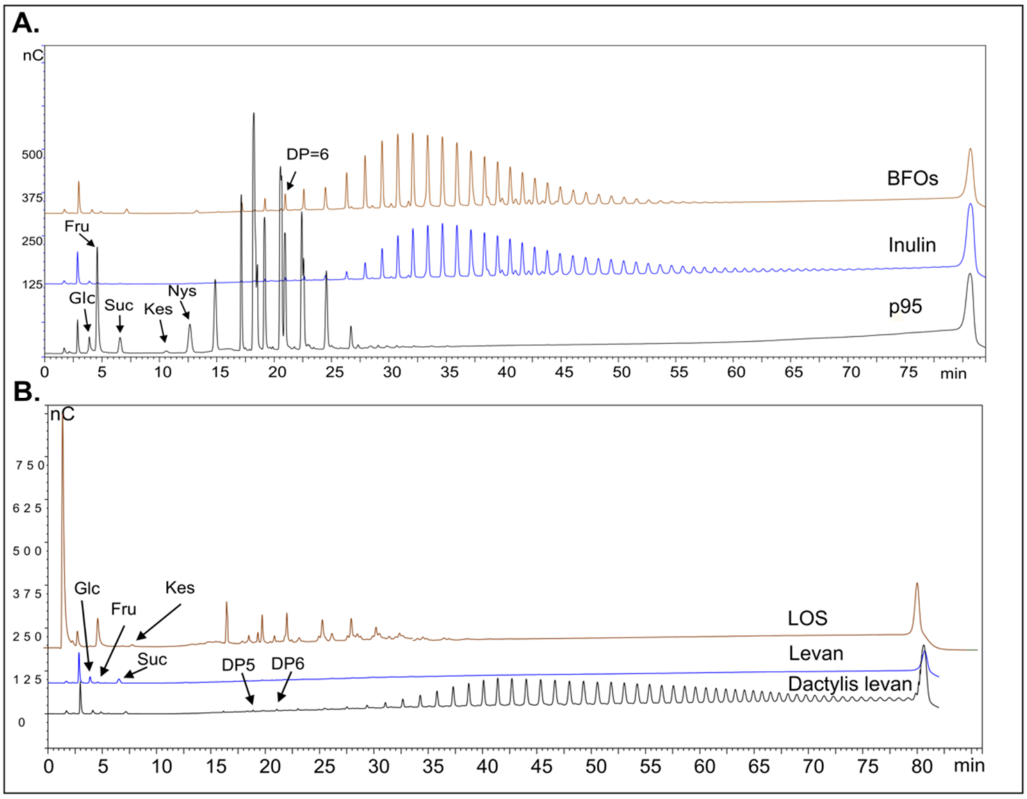

Figure S1. Chromatograms resulting from fructan analysis on HPAEC-IPAD. (**A**) Inulin-type fructans, (**B**) levan-type fructans. Y-axis represents detector response in nanoCoulomb; X-axis represents elution time (min). Glc = glucose; Fru = fructose; Suc = sucrose; Kes = 1-kestose; Nys = nystose.

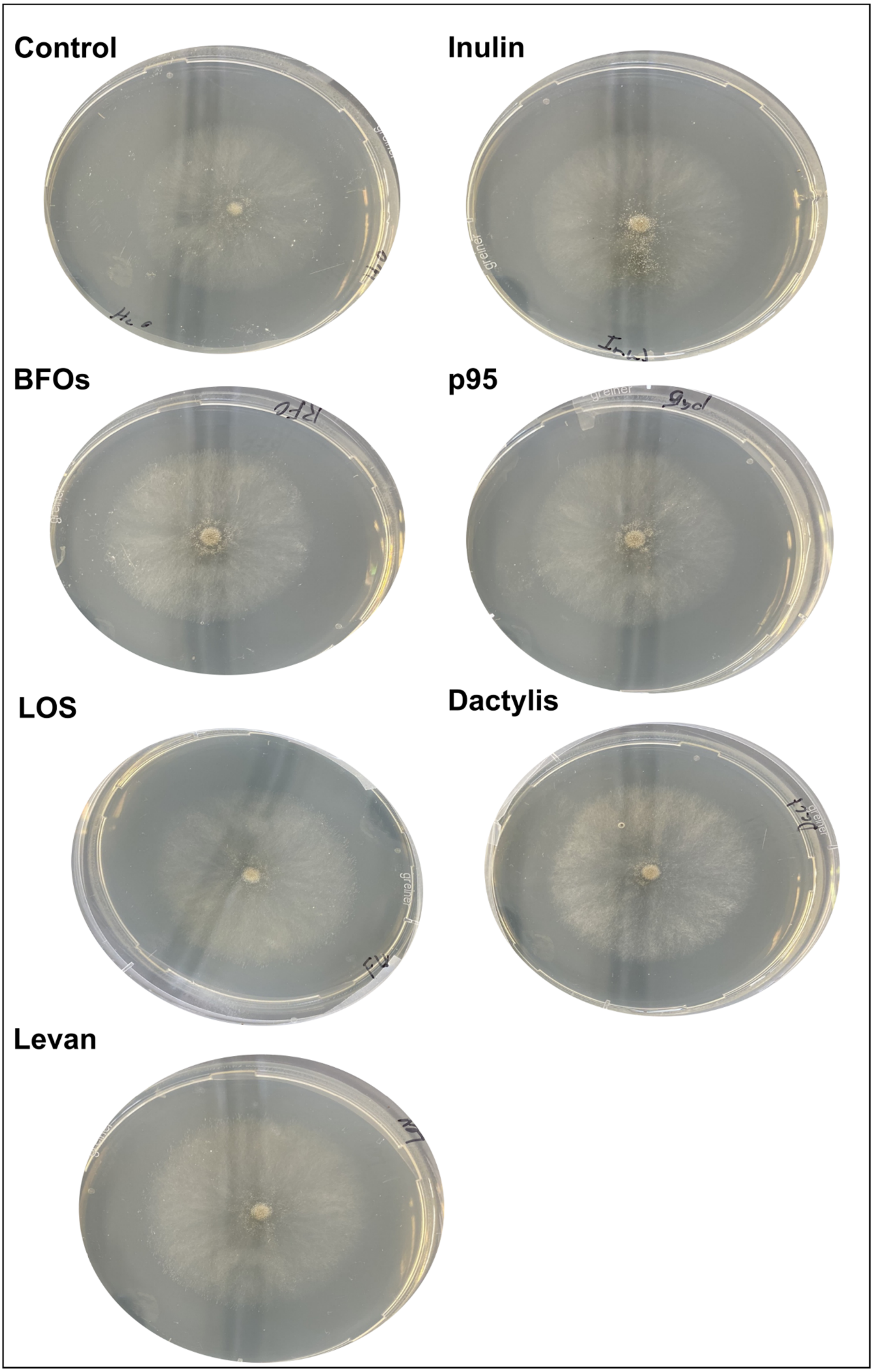

Figure S2. *B. cinerea* growth on 24 g/L PDA plates supplemented with fructans. *B. cinerea* grown for 72 h on PDA plates containing 5 g/L fructan.

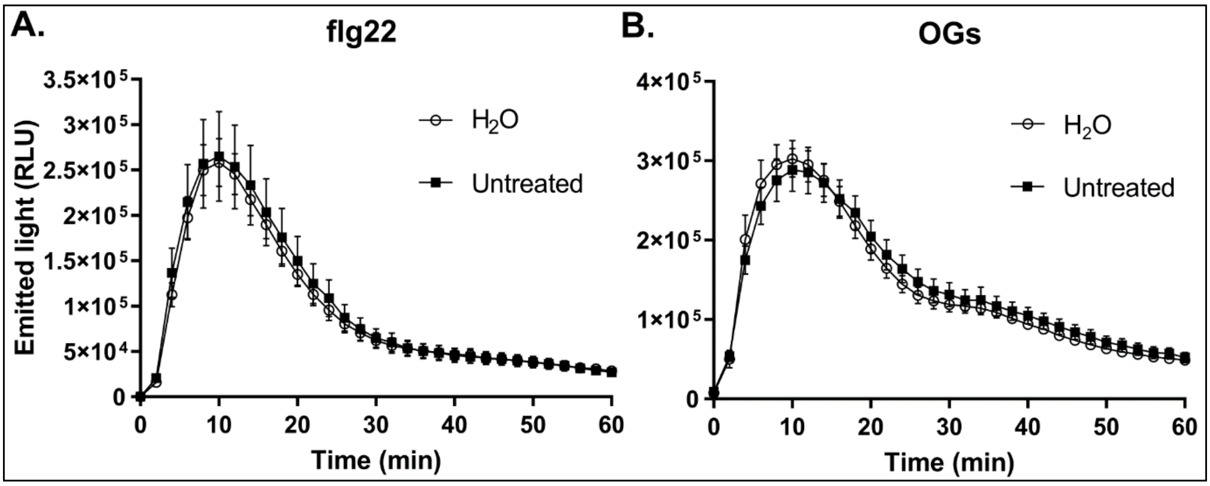

Figure S3. (**A**) flg22- and (**B**) OGs- induced ROS burst in untreated and H_2O treated plants. Untreated *Arabidopsis* or plants pre-treated with H_2O containing 0.0001 % Tween-20 were treated with 100 nM flg22 or 0.2 mg/mL OGs 24 h later to assess the effect of H_2O spraying on ROS burst. Values represent the mean of 8 biological replicates.

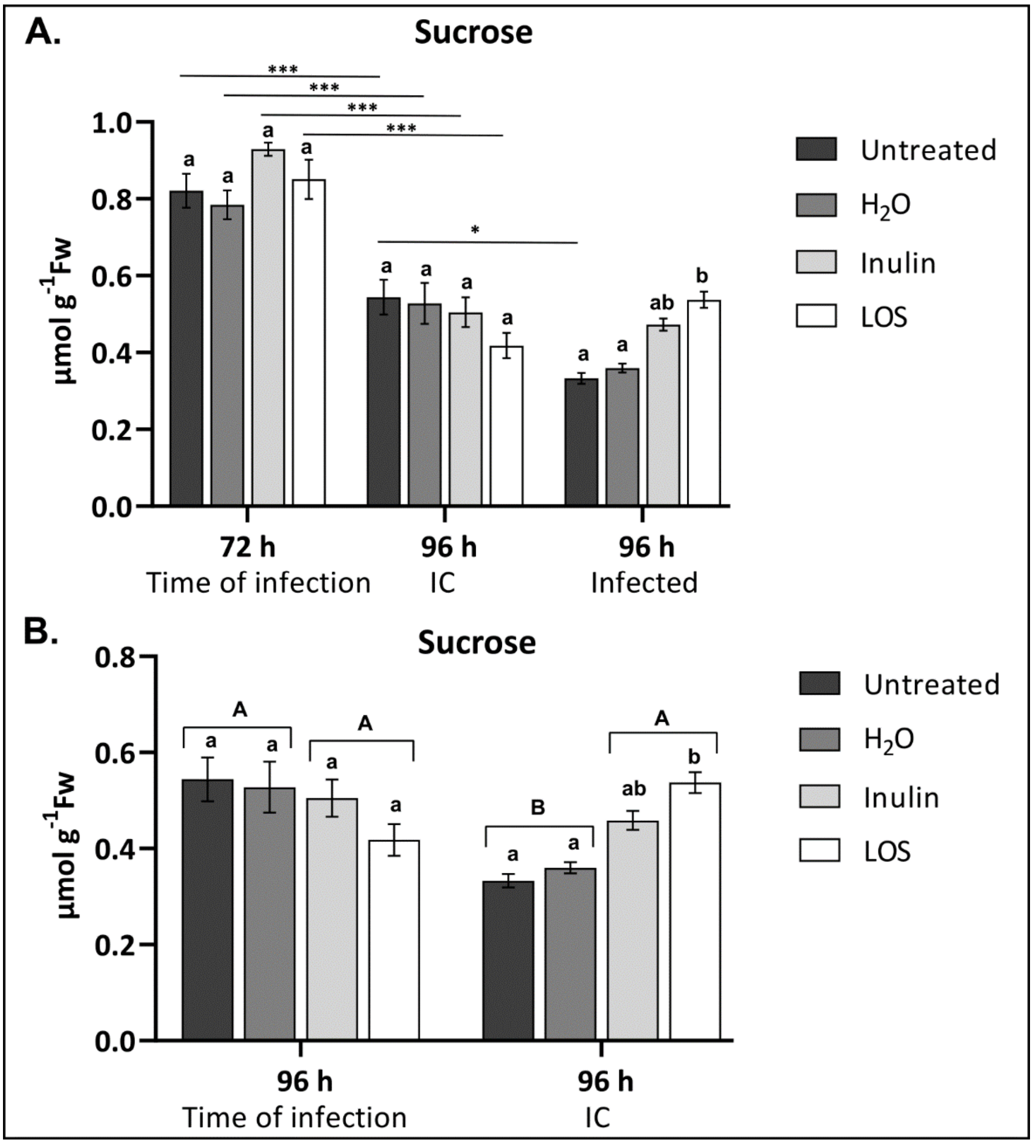

Figure S4. (A) Decrease in Suc content as a consequence of leaf defoliation after 24 h. Statistical significance is indicated by different letters ($p < 0.05$) within the same timepoint or treatment or with an asterisks (*$p < 0.05$; **$p < 0.005$; ***$p < 0.001$) between different timepoints and is based on two-way ANOVA followed by Tukey's multiple comparisons test. (**B**) Effect of fructan priming on maintaining Suc homeostasis after infection. Grouped values of Suc levels for control (untreated and H_2O treated) compared to fructan treated (inulin and LOS) in infection-control (IC) and infected leaves. Statistical significance is indicated by different lower-case letters (*$p < 0,05$) within the same timepoint or treatment or with uppercase between infection-control and infected and is based on two-way ANOVA followed by Tukey's multiple comparisons test.
